# Hydroxychloroquine‐Associated Photosensitivity: A Case Report With Repeat Phototesting

**DOI:** 10.1111/phpp.70130

**Published:** 2026-11-02

**Authors:** John Warner‐Levy, Donna Parkin, Jean Ayer

**Affiliations:** ^1^ Faculty of Biology, Medicine and Health The University of Manchester Manchester UK; ^2^ Department of Dermatology Cutaneous Biology Research Center, Massachusetts General Hospital and Harvard Medical School Boston Massachusetts USA; ^3^ Photobiology Unit, Dermatology Centre Salford Royal Hospital, Northern Care Alliance NHS Foundation Trust, Manchester Academic Health Science Centre Greater Manchester UK

**Keywords:** connective tissue disease, drug‐induced photosensitivity, hydroxychloroquine, minimal erythema dose, photodermatology, photosensitivity, phototesting, scleroderma, UVA, UVB

## 1 Introduction

Hydroxychloroquine is commonly used in the management of inflammatory and connective tissue diseases and may reduce cutaneous disease activity in selected photosensitive conditions [1]. However, its relationship with photosensitivity remains complex, with inconsistent mechanistic and clinical data and reports of hydroxychloroquine‐associated photosensitivity [2, 3, 4, 5, 6]. We describe a case of photobiologically confirmed photosensitivity temporally associated with hydroxychloroquine initiation for scleroderma, characterised by reduced UVB and UVA erythemal thresholds.

Following hydroxychloroquine discontinuation, symptoms and phototesting abnormalities improved substantially, with normalisation of all previously reduced UVA erythemal thresholds and improvement in the remaining reduced 300 nm threshold. Persistent but attenuated broadband UVA provocation responses suggested residual photosensitivity, which may have reflected the underlying connective tissue disease. Written informed consent was obtained for publication of this case and images.

## 2 Case

In May 2024, a 43‐year‐old woman with Fitzpatrick phototype I–II presented with a 2‐year history of marked photosensitivity, reported to have begun shortly after hydroxychloroquine was started for scleroderma. The dose was initially 200 mg daily and later increased to 200 mg twice daily. Her medical history included atopic dermatitis and allergic rhinitis, with no family history of photosensitivity. She reported no history of airborne contact dermatitis and no relevant occupational or environmental exposures. The Dermatology Life Quality Index was 3 over the preceding week and 14 over the preceding year. Regular medications were lansoprazole for more than 5 years, hydroxychloroquine for 2 years, nifedipine for 12 months, mirabegron for 12 months, prednisolone for 8 months and mycophenolate mofetil for 6 months. There was no significant dietary intake of common psoralen‐containing foods or quinine‐containing beverages.

Brief sun exposure triggered symptoms within minutes on hot days and later the same day under cloudy conditions. Episodes were characterised by burning, tingling, pruritus and erythema affecting photoexposed sites, including the forearms, dorsal hands, scalp and forehead, with sharp demarcation from covered skin (Figure 1). Symptoms typically persisted for several days and occasionally lasted longer than 1 week. Light exposure through window glass, indoor lighting and heat alone did not provoke symptoms, although she reported post‐exposure malaise.

**FIGURE 1:**
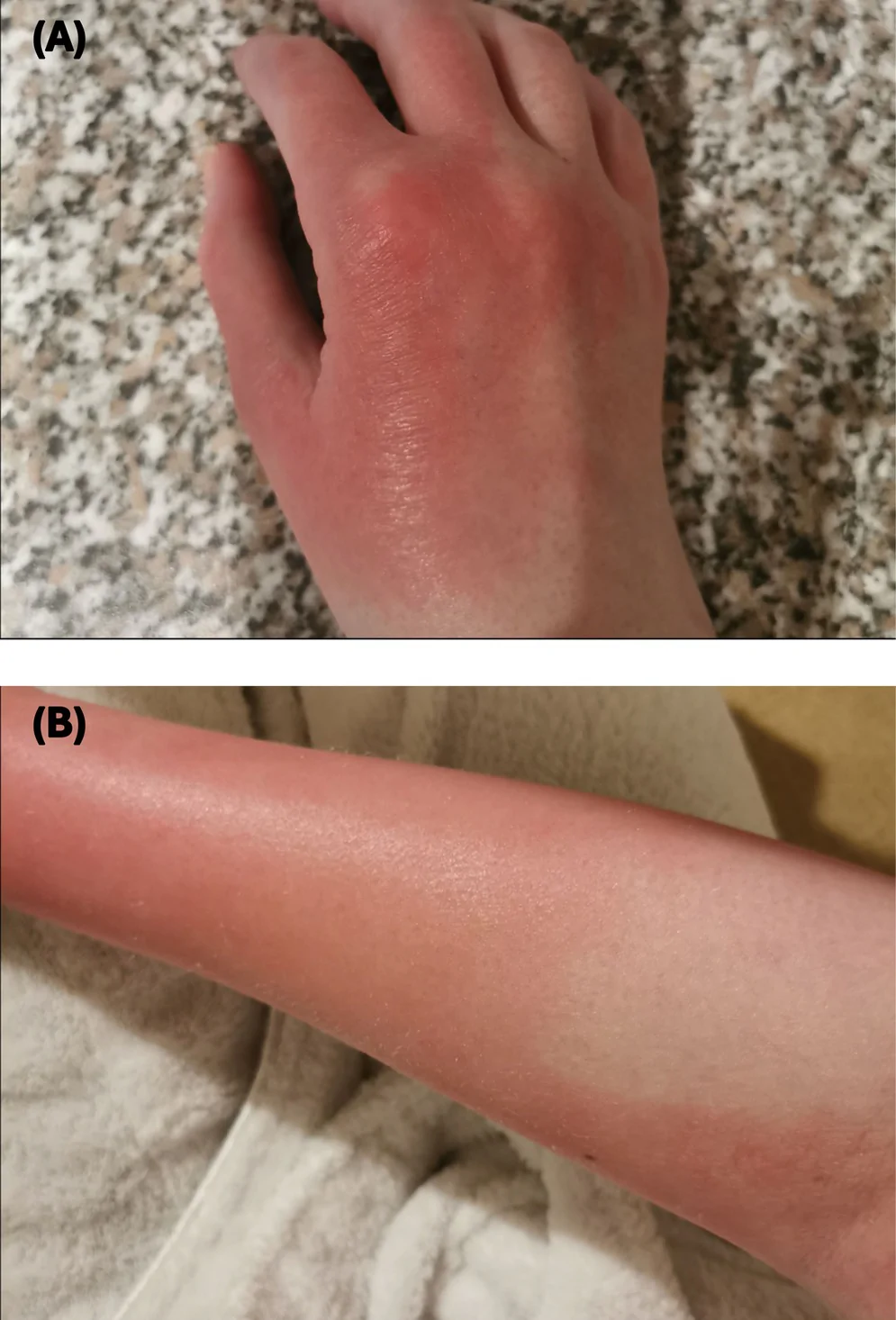
Photodistributed erythema of the dorsal hands (A) and forearms (B), showing sharp demarcation between exposed and covered skin.

Scleroderma‐related manifestations included fatigue, arthralgia, sclerodactyly with elbow contractures and diffuse cutaneous swelling, tightness and waxiness. In view of the temporal association with hydroxychloroquine initiation, absence of scleroderma‐related changes on photoexposed skin and lack of features suggestive of chronic actinic dermatitis, a provisional diagnosis of hydroxychloroquine‐associated photosensitivity was considered. She was prescribed Anthelios SPF 50+ and referred for photoinvestigation.

Monochromator testing, performed using 10‐nm bandwidths, demonstrated reduced minimal erythema doses at 300 nm (7 mJ/cm^2^), 320 nm (1 J/cm^2^), 330 nm (3.5 J/cm^2^) and 350 nm (1.8 J/cm^2^). Thresholds at 370 and 400 nm were not reduced, and no erythemal response was elicited during visible light testing. Broadband UVA provocation at 15 J/cm^2^ induced moderate confluent erythema with scattered papules at 24 h, without an immediate response. Solar simulator testing at 10 J/cm^2^ produced severe confluent erythema with oedema and papulation at 24 h, also without an immediate response.

Photopatch testing to Tinosorb S at 1 J/cm^2^ showed a doubtful reaction at 72 h, consisting of faint macular erythema. Testing to other ultraviolet filters, topical NSAIDs and commercial sunscreens was otherwise negative, including two Tinosorb S‐containing products, Anthelios UVmune 400 SPF 50+ and Anthelios UVmune 400 Invisible Fluid SPF 50+. Conventional patch testing showed no reaction to any product. Photoinvestigation confirmed photosensitivity, characterised by reduced UVB and UVA erythemal thresholds.

Connective tissue disease serology was positive for anti‐SSA and anti‐Ro52 antibodies, with normal vitamin D and IgE levels, and plasma porphyrin screening was negative. Hydroxychloroquine was discontinued due to suspected drug‐associated photosensitivity, while connective tissue disease‐associated photosensitivity remained a differential diagnosis. Prednisolone was discontinued before repeat phototesting to avoid corticosteroid‐related suppression of disease activity, and clobetasol propionate 0.05% ointment was prescribed for flares.

Six months after hydroxychloroquine discontinuation, repeat assessment demonstrated clinical improvement and improved phototesting responses. The minimal erythema dose at 300 nm increased to 10 mJ/cm^2^ but remained reduced, while erythemal thresholds at 320–350 nm normalised, measuring 2 J/cm^2^ at 320 nm, 10 J/cm^2^ at 330 nm and 14 J/cm^2^ at 350 nm. Thresholds at 370 and 400 nm remained normal, and visible light testing remained negative. Broadband UVA provocation (15 J/cm^2^) induced immediate blotchy erythema with whealing, followed by mild erythema and scattered papules at 24 h. Solar simulator testing (10 J/cm^2^) was negative immediately but produced mild erythema with occasional papules at 24 h.

## 3 Discussion

The temporal association with hydroxychloroquine initiation, clinical improvement after withdrawal and normalisation of the previously reduced UVA erythemal thresholds support a hydroxychloroquine‐associated contribution. However, the persistently reduced 300 nm threshold and ongoing, though attenuated, provocation responses suggest residual photosensitivity that may have been related to the underlying connective tissue disease, rather than an entirely drug‐induced process.

This case adds to the limited literature on hydroxychloroquine‐associated photosensitivity. Reported patterns are heterogeneous, including phototoxic, photoallergic, UVB‐predominant and severe photoinduced cutaneous reactions [2, 3, 4, 5, 6]. Here, we document reduced UVB and UVA erythemal thresholds, together with clinical and photobiological improvement following hydroxychloroquine withdrawal. Photosensitivity has also been reported with related quinoline‐based antimalarials, including chloroquine and quinine, supporting consideration of the broader drug class when evaluating photosensitivity [7, 8].

Taken together, these reports reinforce the need to consider hydroxychloroquine‐associated photosensitivity even when the drug is prescribed for a photosensitive inflammatory disorder. In patients with connective tissue disease, symptoms may otherwise be attributed to the underlying condition, while hydroxychloroquine use may falsely reassure against a drug‐associated component. Careful chronology, exclusion of alternative photosensitising exposures, phototesting and repeat assessment after withdrawal may help clarify causality.

## Author Contributions

J.W.‐L. drafted the manuscript and reviewed the literature. D.P. performed the photoinvestigations, contributed to interpretation of the findings and critically revised the manuscript. J.A. supervised the case, contributed to interpretation of the findings and critically revised the manuscript. All authors approved the final manuscript.

## Funding

The authors have nothing to report.

## Conflicts of Interest

The authors declare no conflicts of interest.

## Data Availability

Data sharing not applicable to this article as no datasets were generated or analysed during the current study.
